# Development of a Novel Adsorbent Derived from Olive Mill Solid Wastes for Enhanced Removal of Methylene Blue

**DOI:** 10.3390/ma17174326

**Published:** 2024-08-31

**Authors:** Malak Hamieh, Nabil Tabaja, Sami Tlais, Bachar Koubaissy, Mohammad Hammoud, Khaled Chawraba, Tayssir Hamieh, Joumana Toufaily

**Affiliations:** 1 Laboratory of Applied Studies for Sustainable Development and Renewable Energy (LEADDER), Faculty of Sciences, Doctoral School for Science and Technology (EDST), Lebanese University, Hariri Campus, Hadath P.O. Box 90656, Lebanon; malak_hamieh97@outlook.com (M.H.); nabiltabaja1@gmail.com (N.T.); bachar.kobaissy@ul.edu.lb (B.K.); khaled.chawraba@outlook.com (K.C.); 2 Laboratory of Materials, Catalysis, Environment, and Analytical Methods (MCEMA), Faculty of Sciences, Lebanese University, Hadath P.O. Box 6573, Lebanon; 3 College of Health and Medical Technologies, Alayen Iraqi University (AUIQ), Nasiriyah 64001, Iraq; 4 College of Engineering and Technology, American University of the Middle East, Egaila 54200, Kuwait; sami.tlais@aum.edu.kw; 5 Bahaa and Walid Bassatne Department of Chemical Engineering and Advanced Energy, Faculty of Engineering and Architecture, American University of Beirut, Beirut P.O. Box 11-0236, Lebanon; msh47@mail.aub.edu; 6 Faculty of Science and Engineering, Maastricht University, P.O. Box 616, 6200 MD Maastricht, The Netherlands

**Keywords:** activated carbon, adsorption, Langmuir, Freundlich, Temkin, dyes

## Abstract

Industrial wastewater discharge, tainted with hazardous substances, including dyes like methylene blue (MB) from the textile sector, further emphasizes the need for water treatment to produce safe drinking water. This study explores the potential of olive mill solid waste, an abundant and cost-effective agricultural waste in Mediterranean regions, to yield high-quality activated carbon (AC) with zinc chloride activation for MB adsorption. The activation process, carried out at a modest temperature of 500 °C without the need for an inert atmosphere, resulted in AC with remarkable characteristics, boasting a substantial surface area of 1184 cm^2^·g^−1^ and a total pore volume of 0.824 cm^3^·g^−1^. Extensive characterization of the AC was carried out through a large range of surface techniques. The pH of the solution had minimal influence on MB adsorption, the maximum removal was 95%, which was under slightly acidic pH conditions (5.8), and the adsorbent dose was 0.4 g·L^−1^ for a 50 mg·L^−1^ MB concentration. Equilibrium data pertaining to MB adsorption were subjected to fitting with different models, namely Langmuir, Freundlich, and Temkin. Notably, the Langmuir model exhibited the best fit, revealing a maximum monolayer adsorption capacity of 500 mg·g^−1^ at 25 °C, and the adsorption kinetics closely followed a pseudo-second-order model.

## 1. Introduction

The production of olive oil plays a dominant role in the agricultural industry of Mediterranean countries [1,2], contributing to approximately 94% of the world’s olive oil production [3]. However, this prolific production method generates substantial solid residues known as olive mill solid waste (OMSW) [1]. OMSW is composed of soft pulps, skin, cracked olive stones, and oil residues, and the annual production of OMSW reaches a staggering 2 × 10^6^ tons [4]. The uncontrolled disposal of OMSW into the environment poses a significant threat, potentially leading to severe environmental disturbances. OMSW, due to its antimicrobial properties and high phytotoxicity [5], can hinder seed germination [6], alter soil quality, and impede plant growth [7]. Additionally, the incineration of OMSW releases toxic emissions, raising concerns about atmospheric air pollution [8]. Therefore, it is imperative to implement effective management methods for the treatment of OMSW, not only to mitigate their environmental impact but also to promote the sustainable utilization of resources.

OMSW can find valuable applications across various fields, including energy production through gasification, livestock feed, soil enhancement, the manufacture of briquettes for domestic heating, and pellets for industrial boilers [6,7]. Among these valorization processes, the creation of AC from OMSW emerges as a particularly promising alternative. While commercial AC derived from wood and coal is widely utilized for adsorption in water treatment to remove heavy metals, dyes, pharmaceuticals, and pollutants, its widespread application is hampered by its high cost and regeneration challenges [9]. Currently, researchers are shifting their focus toward sustainable and cost-effective precursors for AC production, with agricultural waste gaining prominence as a favorable option [10,11]. Solid residues from olive mills, in particular, are considered one of the most promising candidates among agricultural waste sources for AC production. Their abundance, especially in Mediterranean countries, coupled with their cost-effectiveness, positions them as a sustainable resource for AC development.

**Figure 1:**
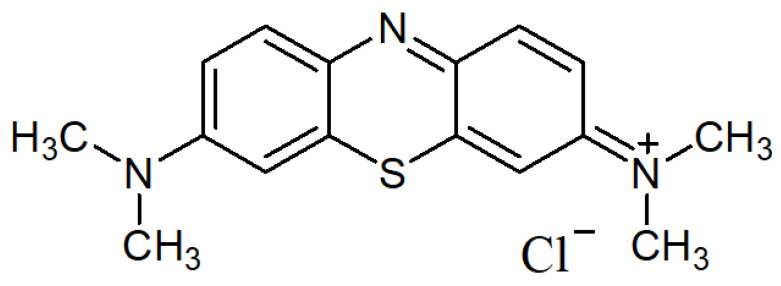
Methylene blue structure.

One of the crucial applications of AC derived from OMSW is its effectiveness in treating water contaminants such as MB [12], a common non-biodegradable water pollutant [13]. MB is a widely utilized substance within the dye industry, finding common application in the coloring of various materials like silk, cotton, wool, and paper [14,15]. In the textile sector, substantial amounts of MB dyes are routinely released into natural aquatic environments, which raises concerns about potential health hazards for both humans and aquatic microorganisms. MB can pose various risks to human health, including respiratory issues, gastrointestinal disturbances, vision impairment, and digestive and psychological disorders. Even at minimal concentrations, the existence of MB in water bodies results in the formation of intensely colored byproducts. Due to its notably high molar absorption coefficient, MB diminishes the penetration of sunlight into the water, consequently reducing oxygen solubility, impairing the photosynthetic processes of aquatic organisms, and diminishing both the biodiversity and the visual appeal of the aquatic ecosystem (Figure 1) [12].

AC production can be achieved via two main methods: physical activation and chemical activation. In the physical activation process, the precursor material is first subjected to carbonization utilizing steam, air, or CO_2_, generally at temperatures surpassing 800 °C [16]. In contrast, chemical activation involves simultaneous carbonization and activation steps. In this method, the raw materials are soaked or treated with an activating agent and subsequently subjected to heating within an inert atmosphere. However, employing an inert medium during AC preparation adds complexity and additional costs to the experimental setup. Commonly used activating agents in chemical activation include ZnCl_2_ [17], H_3_PO_4_ [18], KOH [19], NaOH [20], and K_2_CO_3_ [21]. ZnCl_2_, in particular, serves as an excellent activator due to its dehydrating properties when impregnated with biomass. This property minimizes the formation of tars and elevates both the specific surface area and porosity of carbon materials. Scientists have explored various materials sourced from olive waste and employed a variety of physicochemical treatment methods to develop effective adsorbents for purifying water bodies of contaminants. For instance, activated carbon derived from olive pomace was prepared using chemical activation with H_3_PO_4_ at 500 °C for 2 h under a nitrogen flow, resulting in a recorded BET surface area of 958 m^2^·g^−1^ [22].

In another study, Demiral et al. [23] utilized steam for the physical activation of olive bagasse, resulting in a BET surface area of 718 m^2^·g^−1^ and achieved a 52% removal of Cr(VI) at equilibrium. Conversely, Kula et al. [24] employed chemical impregnation with 20% *w*/*w* ZnCl_2_ followed by carbonization at 650 °C for 2 h under a nitrogen flow using olive stone as the precursor. The resulting AC exhibited a BET surface area of 790.36 m^2^·g^−1^ and around 80% adsorption of Cd(II) ions. The key aim of this study is to utilize OMSW as the starting material for AC production, employing ZnCl_2_ as the activating agent. The objective is to achieve AC preparation at a lower temperature without the need for an inert atmosphere, aiming for cost reduction. Additionally, this research delves into the effectiveness of the synthesized AC in the removal of MB dyes from aqueous solutions. Several physicochemical methods are employed to investigate the specific surface area, optical properties, and structural and textural properties of the prepared AC. The study systematically carries out adsorption experiments to assess how various operational factors, including adsorbent quantity, contact time, initial dye concentration, and pH levels, impact the efficiency of MB removal. Furthermore, kinetics and equilibrium analyses are carried out under specific conditions to investigate and understand the underlying adsorption mechanisms.

## 2. Materials and Methods

### 2.1. Materials

MB was sourced from UNI-CHEM (Mumbai, India). The solubility of MB in water is 43.6 g·L^−1^ at 25 °C, and its pKa value is 3.8. The initial concentrations required for all solutions were prepared by diluting the stock solution with distilled water. ZnCl_2_ and NaOH were purchased from Sigma-Aldrich (Saint Louis, MO, USA). HCl (37%) was obtained from VWR (Radnor, PA, USA). It is important to note that all chemicals used in this study met the analytical-grade quality standards.

### 2.2. Procedure for AC Preparation

#### 2.2.1. Pretreatment of OMSW

The OMSW was gathered following the olive oil extraction process, which typically occurs during the Lebanese olive cultivation season from October to December. To eliminate any extraneous impurities and contaminants, the collected materials underwent a thorough washing with deionized water. Subsequently, these samples were subjected to a drying process in an oven at 110 °C for 24 h. This step is common for all pyrolysis reactions to remove moisture content from the lignocellulose materials. Finally, the residual oil remaining in OMSW was removed by Soxhlet extraction using hexane as a solvent.

#### 2.2.2. Chemical Activation of OMSW

In the chemical activation process, OMSW was chemically activated using a ZnCl_2_ solution as the activating agent. The impregnation method involved stirring pre-treated OMSW samples with ZnCl_2_ solution at room temperature for 24 h (impregnation ratio: mass of ZnCl_2_: mass of OMSW = 2:1). The obtained slurry was subjected to a 24 h drying process in an oven set at 110 °C. Following the drying step, the resulting sample was finely ground to obtain fine particles in preparation for the subsequent carbonization process. The impregnated specimen was enclosed within a sealed crucible and subjected to carbonization in a programmable electric muffle furnace. Carbonization occurred at 500 °C for a duration of 2 h, with a gradual heating rate of 5 °C/min. After carbonization, the resulting AC was permitted to cool, followed by a thorough washing process involving 0.1 M HCl and distilled water to eliminate any excess zinc chloride and mineral residues. Lastly, the AC underwent a 24 h drying period in an oven set at 110 °C before being stored in a container for future use.

#### 2.2.3. Characterization of AC

The characterization of the AC pore structure involved N_2_ adsorption–desorption isotherm measurements at −196 °C using a micromeritics instrument (Gemini VII) (Norcross, GA, USA). Prior to analysis, the AC samples were degassed under vacuum at 200 °C overnight. The specific surface area (S_BET_) was determined using the Brunauer, Emmett, and Teller (BET) method. The overall pore volume, comprising both micropores and mesopores, was calculated by converting the quantity of N_2_ gas adsorbed at a relative pressure of 0.99. The micropore volume was assessed using the t-plot method, while the mesopore volume (V_meso_) was determined by subtracting the micropore volume (V_micro_) from the total pore volume. The phase composition and crystal structure of the AC were analyzed by X-ray diffraction (XRD) in the range from 5 to 55° using a Bruker D8 advance diffractometer (Billerica, MA, USA) with CuKα radiation (λ = 1.5418). Fourier transform infrared spectrometry (FT-IR) was used to analyze the surface chemical functional groups of the AC. FT-IR spectra were collected using a Shimadzu instrument (Kyoto, Japan) with a resolution of 4 cm^−1^, employing the KBr technique and scanning the spectrum in the range of 4000–500 cm^−1^. The zeta potential of the AC was assessed using a Nanoplus HD zeta/nanoparticle analyzer (Osaka, Japan). The determination of the isoelectric point (IEP) involved adding 0.01 g of AC to a set of Erlenmeyer flasks, each containing 50 mL of deionized water. Before introducing the adsorbent, the pH of the mixture was precisely adjusted within the range of 1.5 to 10, achieved by employing either 0.1 M HCl or 0.1 M NaOH. The flasks were then subjected to continuous agitation at 150 rpm for one hour. After this agitation period, approximately 1 mL of the sorbent suspension was extracted for analytical purposes. Five measurements of zeta potential were taken, and the resulting average zeta potential values were plotted against pH across the pH spectrum from 1.5 to 10. The IEP was determined by identifying the pH value at which the electrokinetic potential reached zero. To assess the morphology and structure of the AC, a scanning electron microscope (SEM) with an EDX detector was employed. The SEM analysis was performed using a MIRA3 TESCAN microscope (Brno, Czech Republic).

### 2.3. Batch Adsorption Experiments

#### 2.3.1. Removal Efficiency

The adsorption process was evaluated using batch experiments at room temperature. Various independent variables were investigated, including pH (2–10), adsorbent dosage (0.1–1.2 g·L^−1^), contact time (0–240 min), and initial dye concentrations (50–200 mg·L^−1^). The pH of the solution was changed using HCl or NaOH 0.1 M solution. Each experiment involved adding 50 mL of methylene blue (MB) solution with a known initial concentration to a 250 mL Erlenmeyer flask, followed by the addition of 20 mg of activated carbon (AC). Following this, the flask was positioned on a shaker operating at a consistent speed of 220 rpm. Kinetic studies were conducted by adding a fixed amount of AC (20 mg) to 50 mL of MB solution with different initial concentrations (50–200 mg·L^−1^). Samples were collected at various time intervals (15–240 min) and subsequently centrifuged at 10,000 rpm for 20 min. To determine the removal efficiency, the residual concentrations of MB in all samples were analyzed using a UV–visible spectrophotometer (Hitachi 2001, Chiyoda City, Japan) at a wavelength of 664 nm.

(1) $$\%Removal=\frac{C_{0}-C_{t}}{C_{0}}\times 100$$

#### 2.3.2. Adsorption Equilibrium Studies

The adsorption isotherm tests were conducted at room temperature by introducing a fixed amount of AC (20 mg) into a series of 100 mL Erlenmeyer flasks containing 50 mL MB solutions with varying initial concentrations (50, 100, 150, 200, and 300 mg·L^−1^). The pH of the solutions remained unchanged. The flasks were placed on a shaker and agitated at 220 rpm for 24 h to attain equilibrium. Samples were collected at both t = 0 and equilibrium (24 h) and subsequently centrifuged at 10,000 rpm for 20 min. The residual concentrations of MB in the samples were quantified using a UV–visible spectrophotometer. The quantities of MB adsorbed onto the AC at equilibrium, expressed in milligrams per gram (mg/g), were computed as shown below:

(2) $$q_{e}=\frac{\left(C_{0}-C_{e}\right)}{m}\cdot \;V$$

C_0_ and *C*_e_ (mg·L^−1^): MB initial and equilibrium concentrations;
*V* (L): solution volume;
m (g): adsorbent mass.

## 3. Results

### 3.1. Physico-Chemical Characterization of AC

#### 3.1.1. Surface Area Analysis

**Figure 2:**
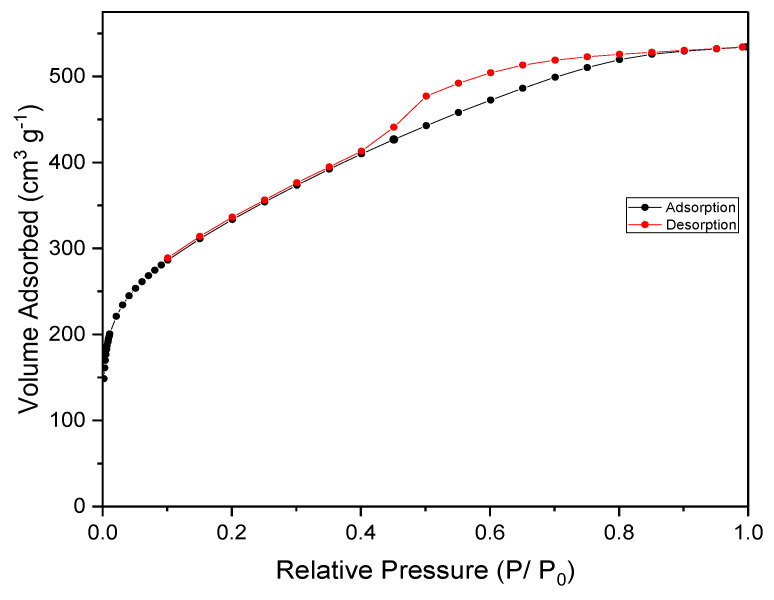
Isotherm for nitrogen adsorption and desorption on AC.

The N_2_ adsorption–desorption isotherm for the prepared AC is presented in Figure 2, revealing important characteristics of its porous structure. In accordance with the classification set forth by the International Union of Pure and Applied Chemistry (IUPAC), the observed isotherm falls into the category of type Ι, indicating the presence of microporous materials [25]. This finding suggests that the AC possesses a network of small pores. Dural et al. obtained comparable isotherms when preparing AC from *Posidonia oceanica* (L) at 600 °C [26]. Likewise, Jaouadi et al. observed a type I isotherm when they prepared AC using olive pomace and phosphoric acid at 500 °C under N_2_ flow for boron adsorption applications [22]. The presence of a mesoporous structure within the AC is evident as the isotherm exhibits a hysteresis loop within the relative pressure range of 0.4 to 0.8. The hysteresis loop corresponds to type IV according to the IUPAC classification and is attributed to capillary condensation occurring in irregular slit-like shaped mesopores. This indicates the existence of larger pores in addition to the micropores, contributing to the overall porosity of the AC [27].

The textural properties of the prepared AC are provided in Table 1, offering valuable insights into its characteristics. The BET surface area (S_BET_) of the AC was determined to be 1184 m^2^·g^−1^. This value surpasses the surface areas reported in previous studies for AC derived from olive waste, despite the fact that the carbonization of OMSW was carried out at a lower temperature of 500 °C and in the absence of a controlled nitrogen medium. This suggests that the preparation method employed in this study resulted in a more porous and higher surface area AC. The AC exhibited micropores and mesopores volumes of 0.055 and 0.769 cm^3^·g^−1^, respectively. Specifically, the micropores (V_micro_) accounted for 6.6% of the total volume (V_T_), while the mesopores (V_meso_) constituted 93.3% of V_T_. The presence of mesopores in carbon significantly reduces the diffusion path length for molecules moving from mesopores to the interior of the carbon material compared to the longer diffusion path when molecules have to traverse directly from the bulk phase to the interior without the assistance of mesopores [28]. Similar results in pore size distribution were observed in the activated carbon (AC) prepared from cotton stalks using ZnCl_2_ activation and investigated under microwave radiation [29]. The textural properties analysis demonstrates that the prepared AC exhibits a high BET surface area and a predominantly mesoporous structure. These characteristics make it suitable for applications requiring the adsorption of larger-sized adsorbate molecules [30].

#### 3.1.2. X-ray Diffraction (XRD)

**Figure 3:**
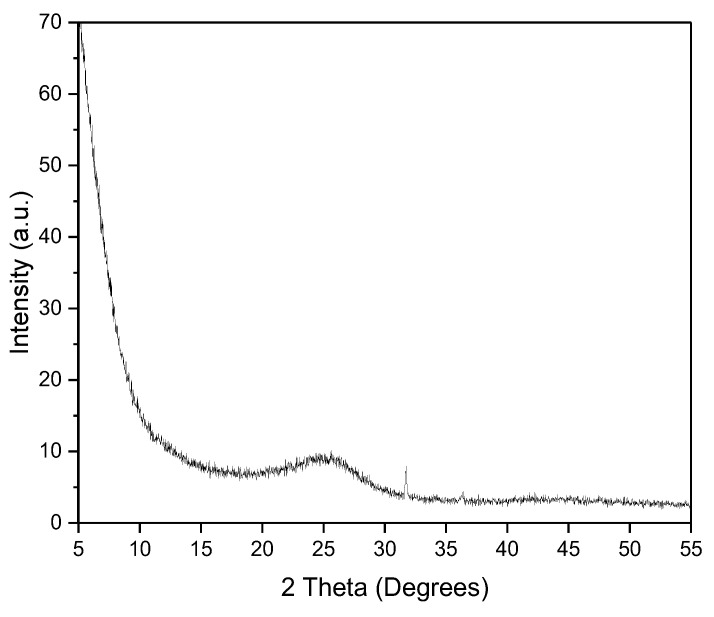
XRD pattern of AC.

The XRD pattern of AC prepared by chemical activation with ZnCl_2_ is presented in Figure 3. The presence of a broad diffraction peak at 2θ = 20–30°, corresponding to the reflection plane (0 0 2), is indicative of the characteristic structure of disordered aromatic carbons [31]. Additionally, the XRD pattern showed a sharp peak at 2θ = 32°, which could refer to the crystalline hexagonal phase of ZnO. The reason behind the appearance of the ZnO peak is the incomplete removal of chemical agents during the washing step. Yang and Lau observed analogous ZnO peaks during their investigation into the preparation of activated carbon from pistachio nut shells using ZnCl_2_ [32]. Additionally, Omri et al. [33] obtained similar broad X-ray peaks for the prepared activated carbon using almond shells for the iodine and methylene blue adsorption.

#### 3.1.3. FT-IR Analysis

**Figure 4:**
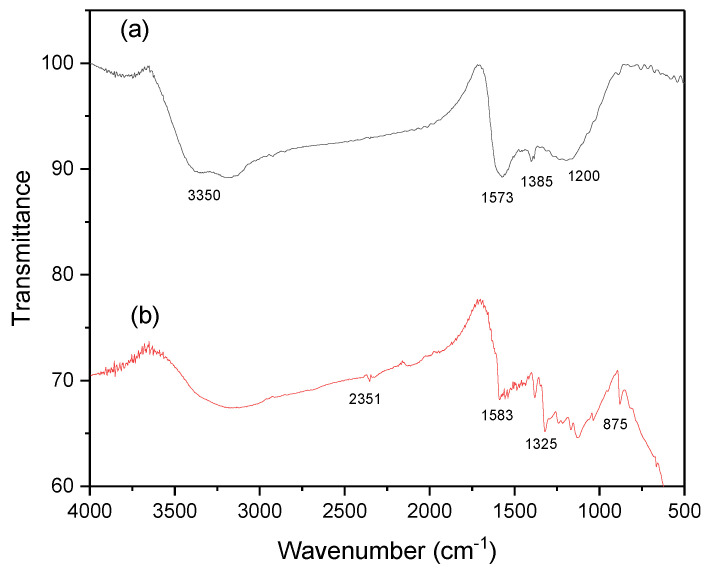
(**a**) FT-IR spectrum of AC and (**b**) FT-IR spectrum of AC after MB adsorption.

Figure 4 presents the FT-IR spectra of the as-prepared AC both before and after the adsorption of MB. In the spectrum representing AC before adsorption, the wide band at 3350 cm^−1^ corresponds to the stretching vibration of hydrogen-bonded hydroxyl groups. Interestingly, this band undergoes broadening and a reduction in intensity following MB adsorption, likely indicative of hydrogen bonding during the adsorption process. Moreover, the peak at 1573 cm^−1^ can be ascribed to the stretching vibration band of C=C bonds in aromatic rings. Notably, this peak experiences a shift after MB adsorption, which can be attributed to π-π stacking interactions. Additionally, there are obvious changes at 1385 cm^−1^ and 1200 cm^−1^, possibly related to alterations in C-H and C-O bonding, respectively.

#### 3.1.4. Zeta Potential Measurements

**Figure 5:**
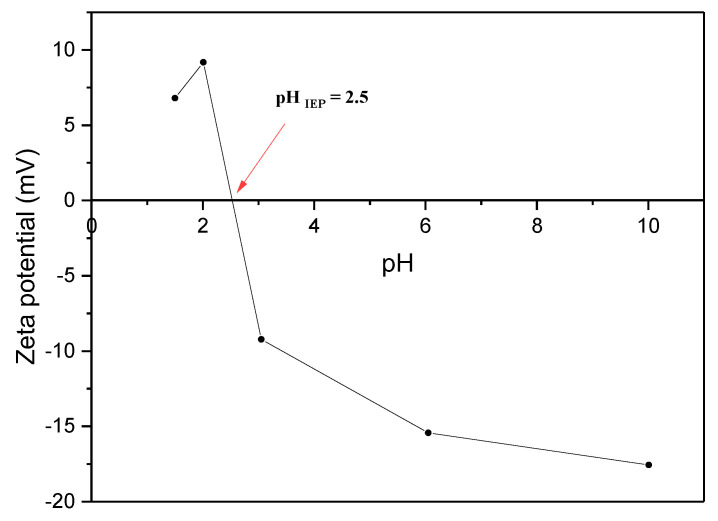
Zeta potential curve of AC at various pH values.

Zeta potential serves as a useful indicator for assessing the acidity or alkalinity of the AC surface. The surface charge of AC is influenced by the types of surface groups and the pH of the solution, which is characterized by the isoelectric point (IEP). Figure 5 illustrates the zeta potential distribution of AC across a pH range spanning from 1.5 to 10, with the observed pH_IEP_ for AC being approximately 2.5. The alteration in the charge of the AC may be attributed to the protonation of functional groups like phenols or alcohols by the excess H^+^ ions. A similar IEP was obtained in the case of AC derived from apricot nut shells prepared using H_3_PO_4_ [34].

#### 3.1.5. SEM-EDX Analysis

**Figure 6:**
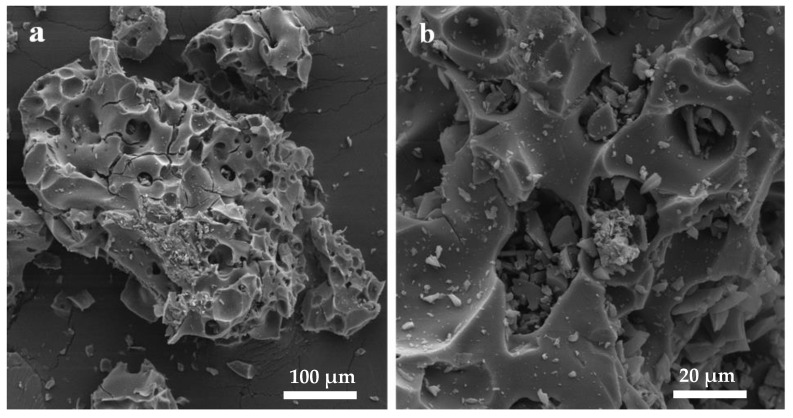
Scanning electron micrograph of AC from OMSW, (**a**) 100 µm, (**b**) 20 µm.

SEM-EDX microchemical analyses were utilized to explore and characterize the complex surface morphology of AC produced from OMSW using ZnCl_2_ as the activating agent. The image reveals a rough surface with numerous cavities of varying sizes and shapes (Figure 6). These cavities can be ascribed to the volatilization of ZnCl_2_ during the carbonization process, resulting in the creation of empty spaces that were previously occupied by ZnCl_2_. Similar voids were observed during the carbonization of silverberry seeds using ZnCl_2_ under ambient air conditions at a temperature of 500 °C for iodine adsorption [31].

Additionally, the surface of the AC shows scattered salt particles, potentially originating from residual zinc salt. This suggests that some traces of zinc compounds remained on the AC surface after the activation process, even after the HCl wash. EDX analysis results indicate that the AC consists of 88.21% carbon (C), 10.72% oxygen (O), 0.62% zinc (Zn), and 0.45% chlorine (Cl). These discoveries provide valuable knowledge into the chemical composition of the AC derived from OMSW after activation with ZnCl_2_. In comparison, olive stone AC, which was prepared at 400 °C, exhibited a textured, densely packed surface with unevenly distributed pores amidst the graphene sheets with C (90.08%), O (6.68%), and phosphorus (P) (3.23%) [35]. EDX analysis for silverberry seeds AC also showed Zn element (0.43%) and Cl (3.10%) in addition to C (96.37%) and sulfur (S) [31].

### 3.2. Adsorption Behavior of MB onto AC

#### 3.2.1. MB Initial Concentration Effect

**Figure 7:**
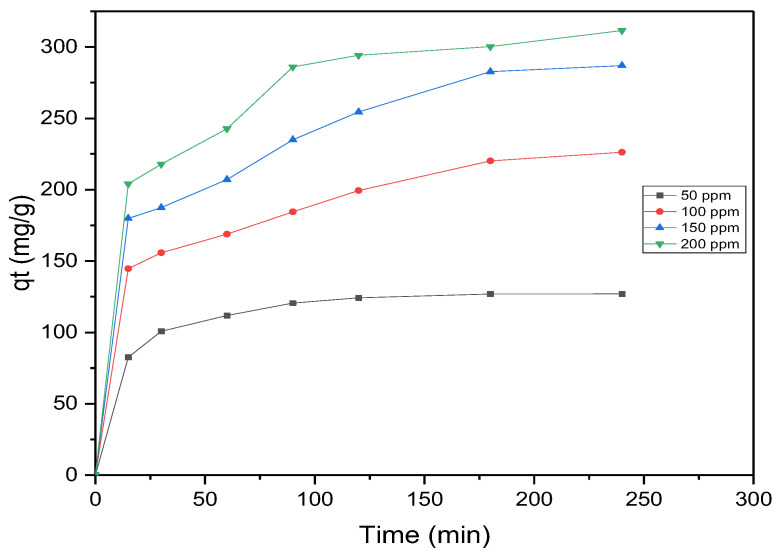
Adsorption time-dependent changes in adsorption capacity for various initial MB concentrations (m_AC_ = 0.4 g·L^−1^, shaking speed = 220 rpm).

The adsorption behavior of MB onto AC was investigated at varying initial MB concentrations (50–200 mg·L^−1^) while keeping the AC dosage constant at 0.4 g·L^−1^. Figure 7 depicts the correlation between the quantity of adsorbed dye (qt) and the duration (t) across various initial MB concentrations. The temporal profile indicates that MB uptake was rapid during the first 30 min, followed by a gradual slowing down as equilibrium was approached. Beyond this point, the removal efficiency reached saturation, signifying no additional removal of dyes from the solution. The initial phase of the sorption process exhibited fast adsorption, primarily due to the abundance of available adsorption sites. As time progressed and equilibrium was reached, the active sites became saturated with dye molecules, resulting in mutual repulsion among adsorbate molecules both on the adsorbent surface and within the surrounding environment. Equilibrium conditions were achieved within 60 min for an initial MB concentration of 50 mg·L^−1^. However, for higher concentrations ranging from 100 mg·L^−1^ to 200 mg·L^−1^, equilibrium was reached within 180–240 min. To ensure complete equilibrium, the adsorption isotherm analysis was conducted over a period of 24 h. The quantity of MB adsorbed per unit mass of AC increased from 128.35 mg·g^−1^ to 311.45 mg·g^−1^ as the initial dye concentration was raised from 50 mg·L^−1^ to 200 mg·L^−1^ (Table 2). The adsorption capacity showed an upward trajectory as the initial MB concentration increased, primarily attributed to the stronger driving force stemming from the elevated initial concentration. As a result, a larger quantity of MB molecules were transferred from the aqueous solution to the surface of the AC, leading to an increased rate of MB removal [34].

#### 3.2.2. Influence of Activated Carbon Dosage on Methylene Blue Removal from Aqueous Solutions

**Figure 8:**
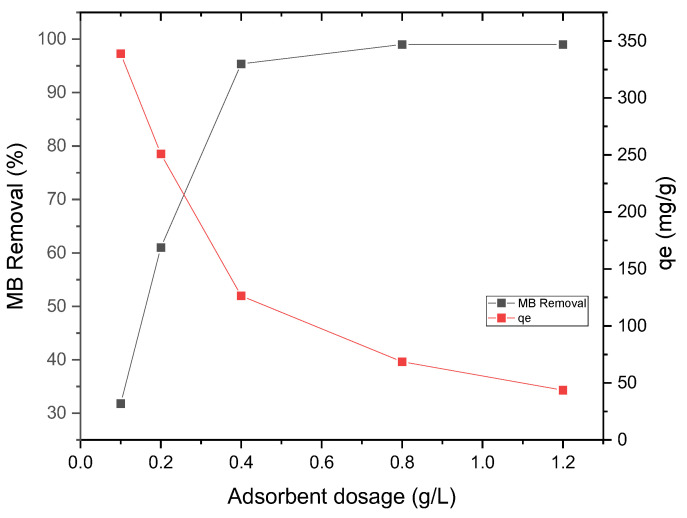
Effect of AC dosage on MB removal (%) ([MB]_0_ = 50 mg·L^−1^, pH = 6.38, contact time = 60 min and shaking speed = 220 rpm).

The influence of AC dosage on the extraction of MB from aqueous solutions was examined by adjusting the quantity of AC from 0.1 g·L^−1^ to 1.2 g·L^−1^ while maintaining a fixed initial MB concentration of 50 mg·L^−1^. Figure 8 illustrates the impact of adsorbent dosage on the percentage removal of MB. The results indicate that as the adsorbent dosage increased from 0.1 g·L^−1^ to 0.4 g·L^−1^, the percentage removal of MB exhibited a significant rise, increasing from 32% to 95% after 60 min of adsorption. This phenomenon can be referred to as the increased surface area and a greater abundance of accessible sorption sites that correspond to higher adsorbent concentrations. However, once the dosage exceeded 0.4 g·L^−1^, the removal of MB remained relatively constant. Consequently, the removal efficiency of MB increased with an elevation in adsorbent concentration until reaching a maximum of 95% at a specific dosage of 0.4 g·L^−1^, beyond which further increases in adsorbent dosage did not result in significant changes in removal efficiency. Furthermore, the data acquired revealed that as the adsorbent mass increased, there was a decline in the equilibrium adsorption capacity (mg·g^−1^). This decline can be attributed to the saturation of adsorption sites during the MB adsorption process. Another contributing factor could stem from the convergence or clustering of adsorption sites, leading to a reduction in the overall accessible surface area for dye adsorption and elongation of the diffusion path. Based on these findings, an adsorbent dosage of 0.4 g·L^−1^ (with a ratio of 1 part MB to 8 parts AC) was selected as the optimal mass for subsequent kinetic experiments, as it achieved the maximum removal efficiency of MB.

#### 3.2.3. Effect of Initial Solution pH

**Figure 9:**
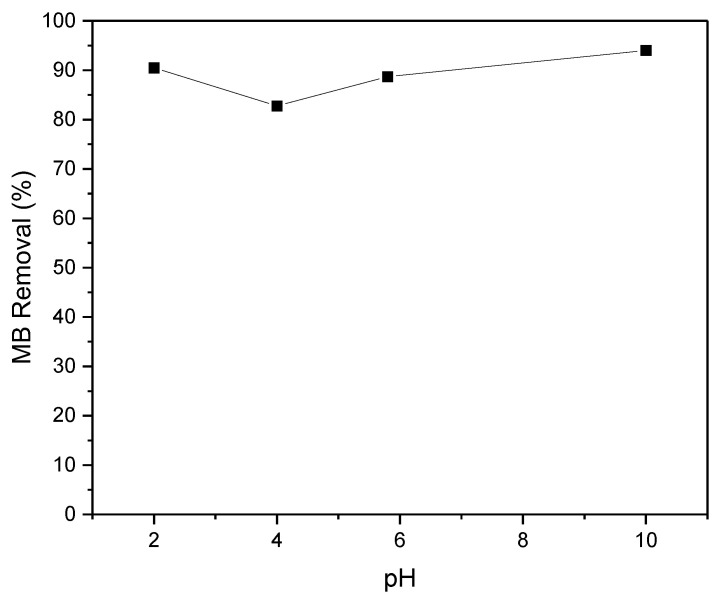
Effect of initial pH solution on the removal of MB (*C*_0_ = 50 mg·L^−1^, m_AC_ = 0.4 g·L^−1^, and contact time = 30 min).

The pH of the solution plays a critical role in governing the adsorption process, affecting both the surface charge of the adsorbent material and the degree of ionization of the adsorbate within the solution. To investigate its impact, experiments were conducted over a pH range of 2 to 10, using an initial concentration of 50 mg·L^−1^ of MB. Remarkably, the results depicted in Figure 9 reveal that the influence of pH on MB removal was relatively insignificant. Even at a low pH of 2 (where pH < pH_IEP_ = 2.5), a substantial removal efficiency of MB (90.5%) was observed. This outcome is surprising, considering the positively charged surface of the adsorbent, which typically hinders the adsorption of cationic dyes due to electrostatic repulsion between the adsorbent and the adsorbate [36]. This phenomenon can be ascribed to the existence of non-electrostatic interactions, notably van der Waals forces and π-π stacking, playing a role in both the adsorption of MB onto the AC and the repulsion between AC particles. These interactions enhance the likelihood of MB adsorption on the non-positively charged sites of the AC. However, the removal efficiency slightly decreased to 82% at pH 4 and then increased to reach 90% at pH 10. In an alkaline setting, the enhanced removal of MB can be attributed to the electrostatic attraction occurring between the negatively charged surface of the adsorbent and the positively charged MB molecules. This electrostatic interaction overcomes the initial repulsion and facilitates the adsorption process, leading to enhanced removal efficiency.

### 3.3. Adsorption Isotherms

The isotherm models serve as valuable tools for gaining profound insights into the adsorption process and elucidating the connection between the adsorbent and adsorbate. These isotherms provide a framework for understanding the interactions between adsorbate molecules or ions and the surface sites of the adsorbent, allowing us to determine the maximum adsorption capacity and optimize the use of the adsorbent. In this study, we employed various mathematical models to analyze the experimental data of equilibrium adsorption. Specifically, we applied the Langmuir, Freundlich, and Temkin isotherm models to comprehensively examine the experimental adsorption equilibrium data.

#### 3.3.1. Langmuir Isotherm

The Langmuir isotherm theory posits that adsorption occurs on a uniform surface, resulting in a monolayer coverage where all adsorption sites share uniformity and possess equivalent energy levels [37]. Also, it assumes that sorption occurs without interaction between the adsorbed molecules on neighboring sites and that there is no migration of adsorbate on the surface. Equation (3) represents the linear form of the Langmuir isotherm:

(3) $$\frac{C_{e}}{q_{e}}=\frac{1}{q_{max}}C_{e}+\frac{1}{q_{max}.K_{L}}\;$$

*C_e_* (mg·L^−1^): equilibrium concentration of MB;
*q_e_* (mg·g^−1^): MB amount adsorbed at equilibrium time per mass unit of AC;
*q_max_* (mg·g^−1^): maximum monolayer adsorption capacity;
*K_L_* (L·mg^−1^): Langmuir constant.

The important characteristics of the Langmuir isotherm is expressed in terms of dimensionless equilibrium parameter (R*_L_*), defined by Equation (4) [38]:

(4) $$R_{L}=\frac{1}{1+K_{L}\times C_{O}}\;$$

*C_O_* (mg·L^−1^): highest initial concentration of MB.

#### 3.3.2. Freundlich Isotherm

Freundlich adsorption isotherm is applicable when dealing with multilayer adsorption on a heterogeneous surface characterized by non-uniform adsorption sites with varying energies of adsorption. The linear representation of the Freundlich isotherm is expressed as follows:

(5) ln⁡qe=1nln⁡Ce+ln KF⁡ 

where the constant *K_F_* is correlated to the adsorption capacity of the adsorbent (mg·g^−1^) and n measures the adsorption intensity. The (*n*) parameter is known as the heterogeneity factor and can be used to estimate whether the adsorption process is chemically (*n* < 1), physically (*n* > 1), or linearly (*n* = 1) favorable. Additionally, the value of 1/*n* reveals normal Langmuir isotherm when 1/*n* < 1 and cooperative adsorption when 1/*n* > 1 [39].

#### 3.3.3. Temkin Isotherm

The Temkin model considers the interplay between adsorbate and adsorbent, acknowledging the indirect relationship between them. This model further assumes that due to these interactions, the adsorption heat for all entities within the adsorbed layer decreases linearly as the coverage increases [34]. The linear form of Temkin isotherm model is expressed as follows:

(6) $$q_{e}=\beta lnK_{T}+\beta lnC_{e\;}$$

where

(7) $$\beta =\frac{RT}{b}$$

*K_T_* (L·mg^−1^): equilibrium binding constant;
*β* (KJ·mol^−1^): Temkin constant;
*R* (8.314 J·mol^−1^·K^−1^): gas constant;
*T* (K): absolute temperature.

**Figure 10:**
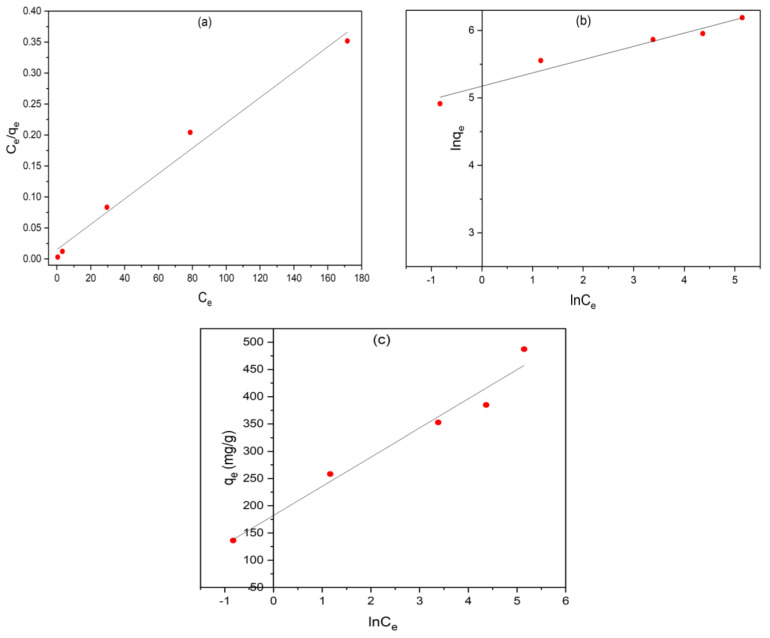
Adsorption isotherm plots for the adsorption of MB onto AC from OMSW: (**a**) Langmuir model; (**b**) Freundlich model; (**c**) Temkin model (*C*_0_ = 50–300 mg·L^−1^, dosage = 0.4 g·L^−1^, pH = 6, equilibrium time = 24 h and T = 25 °C).

The applicability of the mathematical models mentioned above can be validated by comparing the correlation coefficient (R^2^) after the linear plots for each isotherm model (Figure 10). The adsorption parameters of the isotherm models calculated from the experimental data and the values of (R^2^) are listed in Table 3. Based on the results, the Langmuir model has a higher R^2^ value (0.985) than that of the Freundlich and Temkin models (0.959 and 0.969, respectively). This confirms the applicability of Langmuir isotherm, which assumes a monolayer coverage of MB onto AC and the uniform distribution of active sites on the adsorbent surface. For the Langmuir isotherm, the value of R_L_ obtained (0.0245) falls in the range between 0 and 1, implying the favorable adsorption of MB onto AC. Furthermore, the value of n for Freundlich isotherm was found to be higher than unity, which also confirms the favorable adsorption of MB.

Table 4 presents the maximum adsorption capacities (qm) for MB obtained using the Langmuir model, both from prior research and the findings of this study. Notably, our study revealed that the OMSW employed here exhibited a relatively high adsorption capacity of 500 mg·g^−1^, surpassing the capacities of other adsorbents reported in the previous literature. Thus, OMSW could serve as an effective and promising adsorbent for purifying dye-contaminated wastewater.

### 3.4. Adsorption Kinetics

The kinetic study holds significant importance in comprehending the dynamics of adsorption and the underlying mechanism, as it provides insights into the order of rate constants. These studies provide information on MB uptake rate and rate-limiting steps. To understand the mechanism of MB adsorption onto AC prepared from OMSW, most studies used pseudo-first order and pseudo-second order, which are presented below.

#### 3.4.1. Pseudo-First Order

The equation for the Lagergren pseudo-first-order rate is as follows:

(8) $$\log\left(q_{e}-q_{t}\right)=logq_{e}-\frac{K_{1}t}{2.303}\;$$

**Figure 11:**
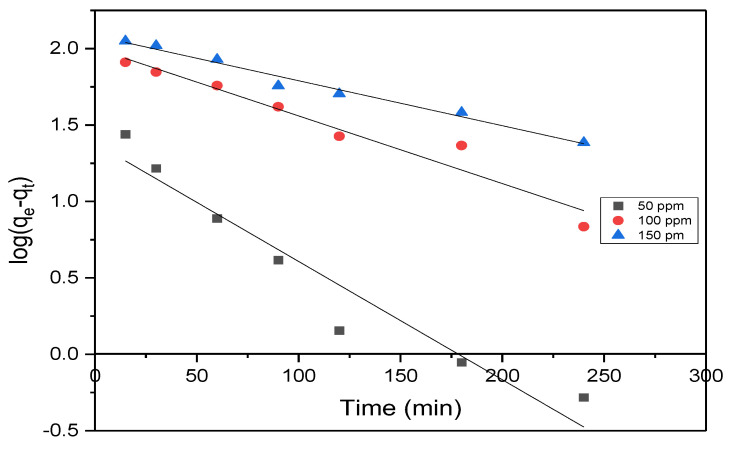
Pseudo-first-order model for MB adsorption on AC.

*K*_1_ and *q_e_* values were calculated using the slope and intercept of log (*q_e_* − *q_t_*) versus t plot (Figure 11). The rate constants, experimental equilibrium uptake, and corresponding correlation coefficients (R^2^) for all initial concentrations tested are shown in Table 5. As can be revealed, the R^2^ values of the pseudo-first-order model for all different initial concentrations were above 0.9, but the calculated *q_e_* values did not match with the experimental *q_e_* values, and this confirms that the adsorption of MB on AC from OMSW did not follow the pseudo-first-order kinetic model.

#### 3.4.2. Pseudo-Second Order

The pseudo-second-order equation is as follows [50]:

(9) $$\frac{t}{q_{t}}=\frac{t}{q_{e}}+\frac{1}{K_{2}{q_{e}}^{2}}\;$$

**Figure 12:**
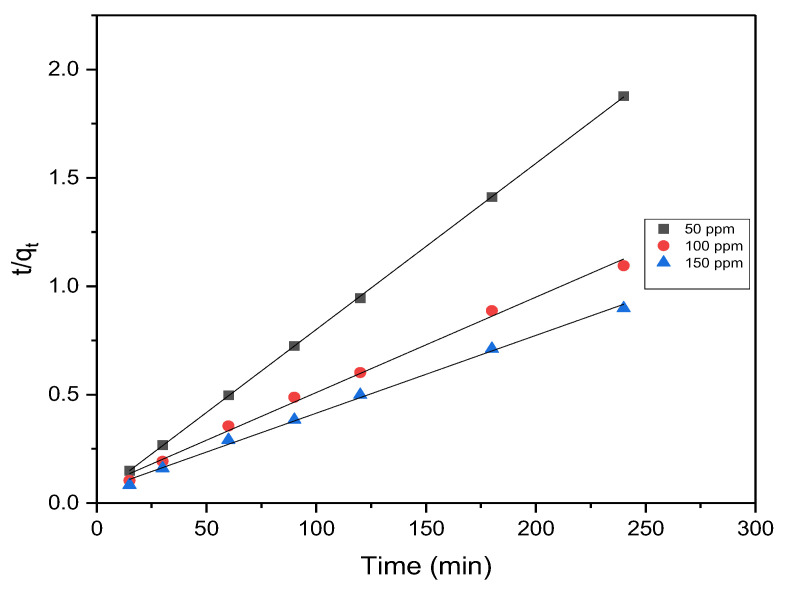
Pseudo-second-order plot for MB on AC.

When plotting *t*/*q_t_* against *t*, it results in a linear relationship, as illustrated in Figure 12. The corresponding R^2^ values, calculated for the pseudo-second-order kinetic model across all initial concentrations, are provided in Table 5. It is notable that the theoretical adsorption capacity, as calculated by the pseudo-second-order model, closely approximated the experimental values for all initial concentrations. Furthermore, the pseudo-second-order model consistently exhibited higher R^2^ values in comparison to the pseudo-first-order model, affirming a strong agreement between the adsorption process of MB onto AC from OMSW and the pseudo-second-order kinetic model. The excellent match with the pseudo-second-order model underscores that the adsorption rate relies on interactions between both the adsorbent and adsorbate.

### 3.5. Adsorption Mechanism of MB

The investigation of the adsorption mechanism governing the interaction between methylene blue (MB) and activated carbon (AC) surface is of utmost importance. As discussed in the previous sections, we can conclude from the equilibrium and kinetic data the existence of both physical and chemical adsorption processes. This was confirmed by Fourier transform infrared (FT-IR) spectroscopic analysis performed after MB adsorption. Based on the aforementioned results of FT-IR before adsorption, the surface of AC contains oxygen-containing functional groups, such as carboxyl, alcohol, ester, lactone, and phenol. The reduction and broadening of the stretching vibration band of the OH group at 3350 cm^−1^ in the FT-IR spectrum of MB after adsorption confirms the physical interaction between MB and AC. Chemical interaction between MB and AC is affirmed by the band shifts observed at 1537 cm^−1^, 1385 cm^−1^, and 1200 cm^−1^. The planar conformation of MB, featuring an aromatic ring, facilitates π-π bond formation between its π electrons and those on the AC surface, thus promoting MB adsorption onto AC. Moreover, the electrostatic interactions are validated by the variation in solution pH. At pH values above the isoelectric point (IEP) of AC (pH 2.5), electrostatic attractions dominate due to the cationic nature of MB and the negatively charged AC surface. Conversely, at pH values below the isoelectric point, MB molecules undergo adsorption through hydrogen bonding with the oxygen-containing functional groups (COOH and COH) on the AC surface. Additionally, intermolecular van der Waals forces play a role in the MB-AC adsorption process. These mechanistic insights provide a comprehensive understanding of the pH-dependent MB-AC interaction, with implications for diverse applications, including environmental remediation and wastewater treatment.

### 3.6. Adsorption of Different Types of Pollutants

The efficiency of AC in the adsorption of various pollutants, including dyes, such as methyl orange (MO) and rhodamine B (RhB), as well as pharmaceuticals, tetracycline (TC), and ciprofloxacin (cipro) was studied. The results are depicted in Table 6. The results reveal excellent adsorption of both pollutants and dyes within only 30 min reaction time. The adsorption percentages were 95%, 97.57%, 87.19%, and 97.52% for MO, RhB, TC, and cipro, respectively. Therefore, our prepared AC from OMSW could be effectively applied on a large scale for treating wastewater from industrial and hospital discharges.

## 4. Conclusions

In this study, activated carbon (AC) was successfully produced from agricultural olive mill solid waste (OMSW) without the need for an inert N_2_ medium, offering a low-cost and highly efficient adsorbent. The prepared AC exhibited a high surface area of 1184 m^2^·g^−1^ and a porous structure filled with cavities, providing ample adsorption sites for dye removal. The investigation of pH’s impact on the adsorption capacity revealed that its impact was relatively limited due to the combined contributions of electrostatic and non-electrostatic interactions, as well as the surface chemistry of the prepared AC. This implies that the AC can be employed effectively across various aqueous mediums for dye removal applications. The adsorption capacity was found to increase with both the initial dye concentration and the dosage of the adsorbent. The equilibrium data aligned well with the Langmuir model, indicating a maximum MB adsorption capacity of 500 mg·g^−1^. The calculated value of R_L_ (the dimensionless separation factor) was 0.0245, further confirming the favorable adsorption of MB onto the AC. The kinetics of the adsorption process exhibited a pseudo-second-order kinetic behavior, highlighting its inherent rapid and efficient nature. This observation indicates that the rate of adsorption depends on both the concentration of the AC and the initial concentration of MB.

Overall, the results demonstrate that OMSW serves as a suitable precursor for producing high-quality AC with exceptional adsorption properties. The findings support the utilization of OMSW-derived AC as a potent adsorbent for the efficient removal of challenging contaminants from water sources.

## Figures and Tables

**Table 1:** Textural properties of AC derived from OMSW.

Material	S_BET_ (m^2^·g^−1^)	V_T_ (cm^3^·g^−1^)	V _micro_ (cm^3^·g^−1^)	V _meso_ (cm^3^·g^−1^)
OMSW	1184	0.824	0.055	0.769

**Table 2:** Variation of q_e_ with increasing MB concentration.

MB Concentration (mg·L^−1^)	q_e_ (mg·g^−1^)
50	128.35
100	226.11
150	286.94
200	311.45

**Table 3:** Parameters of isotherm models and correlation coefficients for MB adsorption onto AC derived from OMSW.

Type of Isotherm	Parameters	Values
Langmuir	q_m_ (mg·g^−1^)	500
	K_L_ (L·mg^−1^)	0.132
	R_L_	0.0245
	R^2^	0.985
Freundlich	K_F_ [(mg·g^−1^)(mg^−1^)^1/n^]	176.65
	1/n	0.197
	R^2^	0.959
Temkin	β	53.45
	K_T_	30.32
	R^2^	0.969

**Table 4:** Assessment of MB adsorption capacities in various ACs.

Adsorbents	S_BET_ (m^2^·g^−1^)	Activating Agent	q_m_ (mg·g^−1^)	Adsorbent Dose (g·L^−1^)	pH	Reference
Tea waste	850.58	H_3_PO_4_	238.1	0.15	-	[40]
*Posidonia oceanica* L.	1483	ZnCl_2_	217.39	10	6.5	[26]
*Cashew nut* shells	1871	ZnCl_2_	476	1.25	7	[41]
Soybean dregs	643.58	ZnCl_2_	255.1	0.5	6	[42]
*Bamboo*	1896	KOH, CO_2_	454.2	1	7	[43]
Date stones	1045.61	ZnCl_2_	369.38	0.5	7	[44]
*Coconut husk*	1940	KOH, CO_2_	434.78	1	7	[45]
Orange peel	1104	K_2_CO_3_	382.8	1	6	[46]
Pomelo skin	1335	NaOH	501.1	1	6	[47]
*Spathodea campanulata*	1054.08	H_3_PO_4_	86.207	2	9	[48]
Rose seeds	1265	ZnCl_2_	288.01	4	-	[49]
Banana stem	837.453	H_3_PO_4_	101.01	2	7	[50]
*Vitex doniana* seed	933	ZnCl_2_	238	1	-	[51]
Peach stone	1298	H_3_PO_4_	412	1	-	[52]
Olive mill solid waste	1183	ZnCl_2_	500	0.4	6.38	This work

**Table 5:** Kinetic parameters for pseudo-first and -second order for adsorption of MB on AC.

C_0_ (mg·L^−1^)	q_e exp_ (mg·g^−1^)	Pseudo-First Order			Pseudo-Second Order		
		K_1_ (min^−1^)	q_e_ (mg·g^−1^)	R^2^	K_2_ (g·mg^−1^. min^−1^)	q_e_ (mg·g^−1^)	R^2^
50	128.35	0.0178	24.06	0.9345	0.0017	129.87	1
100	226.11	0.0101	100.57	0.9509	0.00027	227.27	0.9952
150	286.94	0.0067	121.41	0.9813	0.00023	278.55	0.9968

**Table 6:** Adsorption percentage of different pollutants using AC (*C*_0_ = 20 mg·L^−1^, dosage = 0.4 g·L^−1^, and time = 30 min).

Pollutants	Adsorption (%)
MO	95
RhB	97.57
TC	87.19
Cipro	97.52

## Data Availability

The original contributions presented in the study are included in the article, further inquiries can be directed to the corresponding authors.
